# Pathology, Causes, Course and Treatment of Rheumatism

**Published:** 1873-04

**Authors:** James S. Whitmire


ARTICLE III.—*Pathology, Causes, Course and Treatment of Rheumatism.* By JAMES S. WHITMIRE, M.D. Read before the Woodford County Medical Society, Metamora, Ill., Tuesday, Jan. 7, 1873.

MR. PRESIDENT, AND GENTLEMEN OF THE ASSOCIATION:

Having been appointed at our last sitting to make a special report upon rheumatism, its pathology, causes, course and treatment, I would beg leave to submit the following for your consideration.

In the first place, rheumatism is one of the oldest diseases known and recognized by the profession as a distinct disease, manifesting a peculiar character of inflammation, which is confined in a great majority of cases exclusively to the fibrous textures or membranes of the animal organism. It may be considered as possessing two distinct stages, the acute and chronic. The grade between these two stages may be reckoned as sub-acute, and partakes of the nature of both the acute and chronic stages, and hence it cannot be considered a distinct stage. The acute stage of rheumatism usually occurs as the primary disease, and its decadence into the sub-acute or chronic stage is simply a subsidence of the original attack into the latter stage. It is true, however, that, at the onset of the disease, we may have a sub-acute condition, and if not properly treated, or through indiscretion, this condition may develop into the well marked acute disease or subside into the chronic form. This is just as likely to occur as though it had been acute in the beginning, and probably more likely, from the fact, that, as the pain is less severe than in the acute stage, it is more liable to be neglected.

Rheumatism is an inflammation, but it is peculiar in its characteristics, affecting only one particular or special tissue—the fibrous—and this inflammation, though affecting primarily the fibrous tissues, may extend to other tissues of the body from contiguous sympathy, and therefore rheumatic inflammation may be present, or take place in any part of the animal organism where this (fibrous) tissue is present. We may therefore expect, most frequently, to see it seizing upon and affecting those parts which are the most abundantly supplied with this tissue; hence the large joints, tendons and pericardium are the most frequent recipients of its unwelcome visitations. Rheumatic inflammation is a peculiar as well as a specific disease. It is peculiar, because there is no tendency, while it is confined to its proper tissue, to suppuration or gangrene, a not infrequent result following ordinary inflammations of any or all the other tissues of the body. It is specific, because it has little or no tendency to affect any other but the fibrous tissue; but when rheumatic inflammation extends to other tissues from contiguity or sympathy, the character of the inflammation may or may not be changed, and its character may be the same, and the termination the same, as though it had arisen from any other cause, and may, consequently, be characterized by the usual phenomenon of ordinary inflammations, viz: pain, heat, redness and swelling, and its terminations are, consequently, by resolution, with or without effusion, by suppuration or gangrene, according to the circumstances of the grade, or intensity of the inflammation.

While the grade of rheumatic inflammation in the fibrous tissues may be ever so acute, and the intensity of the pain correspondingly great and persistent, I believe there are few if any cases on record that have terminated in either suppuration or gangrene; while, on the other hand, it may terminate as other inflammations, in resolution with or without effusion, and hence its peculiarity and specific character.

The object of this paper is, not so much to institute a more thorough investigation into the course of this disease, or the character of the inflammation attending it—these being sufficiently well understood, and treated of at length in all our standard works—as it is to invite the attention of the profession to the idea or theory, originally suggested and acted upon in the treatment of this disease, by the late Prof. J. K. Mitchell, of Philadelphia, which was, in substance, this: that rheumatism is a constitutional disease, the inflammation being brought about and maintained in consequence of functional derangement of the spinal cord; which derangement is produced by either active or passive congestion, or perhaps inanition of that important appendage to the great center of the nervous system.

It is only necessary, in this place, to mention the conditions necessary or most likely to disturb the functions of the spinal cord, and therefore the circumstances most likely to produce rheumatic inflammation; these are, exposure to the vicissitudes of the weather without sufficient clothing; heat, cold and moisture, wet feet, and the like; and these may not be the direct causes of the disease—they are most likely, if not certainly, the exciting cause. The view held by the greatest number of pathologists is, that there is a morbid material always present in the blood, in this disease; this materies morbi has been supposed by many to be uric acid, but chemical analysis by our best qualified observers has failed to establish its presence in a greater quantity than in health, while in gout—an analogous disease—its presence in a morbid quantity has been clearly demonstrated. "Others," says Prof. Flint, "have adopted the supposition of Prout, that the morbid principle is lactic acid. The majority of pathologists, at the present moment appear to regard this supposition as probably correct, and it is generally accepted as a rational basis of treatment." "Lactic acid is supposed to be formed during the destruction of sugar in the lungs in health; it may be conjectured, therefore, that its presence in the general circulation depends on circumstances which either occasion its undue formation, or which interfere with its entering into combinations leading to its disappearance. It has also been conjectured that lactic acid is formed in the decomposition of the gelatinous and albuminous substances." This now brings us directly to the point at issue. Assuming, therefore, the lactic acid hypothesis to be true, the query naturally arises, may not this disintegration of those tissues, and the consequent formation of lactic acid, be brought about and even facilitated by the supposed functional derangement of the cord? And may not the failure of the acid to form new combinations in the lungs, and thereby preventing its elimination, be consequent upon the same functional disturbance? These are questions that naturally arise; and while we are conceding the acid theory of this pathological condition of the system—which is the acknowledged hypothesis—may we not, rationally, look for the cause in the direction of the *great centers*, that preside over and govern the nutrition and decay of the economy, while the elimination of all morbific materials from the blood is dependent upon the functional integrity of the same governing cause? Most certainly! is the only rational answer that can be made.

From time immemorial, the practice of the profession, in this disease, has been to a greater or less extent empirical; it has always, however, been governed by the greatest light and the most mature judgment that could be brought to bear in the premises; latterly however, since the co-ordinate branches of science—chemistry and pathology—have been levied upon by the profession, and made to yield up their treasures for the benefit of mankind, we no longer have to grope our way in the dark, theorizing and experimenting in order to arrive at conclusions, but at once apply principles and knowledge where empiricism was once the rule; hence, in the treatment of the disease under consideration, the professional mind has undergone a great change, and what was once considered a formidable disease, has become, under the light of science, one of the most easily managed of any in the catalogue of human diseases.

It has ever, until recently, been a query in the minds of the ablest men of the profession, why this peculiar inflammation should be so erratic in its course, and so stubborn and persistent in its duration, notwithstanding the great variety of means at our command which are used to allay the pain and stay the progress of the disease. The well-known fact, that rheumatism affecting one limb, is more likely, in its peregrinations over the body, to light upon the other on the opposite or corresponding side, or persistently to remain on the same side, manifesting no disposition to cross the median line, would seem to indicate of itself that there must be some central organic derangement, if not absolute disease, that decides the position and locality of the disease. The economy depends for its healthy vitality upon these great centers, hence those portions of the organism become diseased when the functions of organic life from which they receive their healthy action are disturbed, and recover them again so soon as those functions are restored to their normal condition. This regularity in the transition of this disease from one part, or joint, to a corresponding one of the same or opposite side, is the rule; but there are exceptions not unfrequently noticed, such as its sudden transmission to the heart or its investing membranes, yet, true to its steady specific character, even in this instance it makes its appearance first in the fibrous tissues, and then may extend to the serous membranes from contiguous sympathy, through the basement membrane which is areolar, or compound fibrous tissue. It is therefore not to be wondered at, that the pleura and peritoneum are subject to the occasional invasion of this malady, because of the compound fibrous structure of their basement membranes. Now the easiest and most rational conclusion respecting the cause of this uniformity in its migratory tendency, is, that the cause of the disease rests in, and is governed by functional derangement of the spinal cord, and this being the seat of organic life from which the whole animal economy receives its vitalized support, we would naturally look to that source to account for any disturbance of these functions, and more especially those so commonly manifested in corresponding or co-ordinate portions, as well as corresponding tissues of the body.

Again, if the rheumatic inflammation has proven so unyielding as to resist all known remedies and methods of treatment, and gone on to the extent of producing confirmed deformity, we nearly always, if not quite universally see that deformity in exact harmony with its acknowledged laws of co-ordination, consequently corresponding articulations and tissue, either on the same or opposite sides, are affected. Therefore taking for granted that the inferences drawn from these circumstances are true, the following questions would naturally arise: What is the nature of the functional derangement of the spinal cord? And what are the predisposing or exciting causes of such derangement? The predisposing or exciting cause of the disease is sufficiently set forth and commented upon in our standard works on theory and practice, under the head of causes or influences favorable to the development of rheumatism, and therefore they need but a casual mention in this paper; however, it may be well enough in this place to add our testimony to the now general opinion, that there is a rheumatic diathesis which may be either hereditary or acquired, the subjects of which are more prone to attacks of this disease than those who are exempt from this hereditary or acquired taint, hence a very slight exciting cause, in one possessing this predisposition to the disease, will develop the malady in its most aggravated form, whereas the same circumstances would be passed by without exciting the least irregularity in the health of one exempt from this predisposition.

That the predisposition to this disease is inherited, I have not the least doubt; but, because an individual has inherited the taint—if it may be so called—it is no evidence that he will certainly, at some time in life, be afflicted with rheumatism, because an exciting cause may never be presented to him, nevertheless, the predisposition is there, and will remain with him. This we see exemplified in other acknowledged hereditary diseases, such as gout, asthma, tuberculosis, scrofula, etc. Most of the members of such families will sometimes be exempt for one or two generations, and then in the third, perhaps, it will be developed with the most deadly malignancy.

As to the question in regard to the nature or cause of the derangement of the cord, it is more easily asked than answered, from the fact that seldom or never does any person die of this disease, excepting, perhaps, now and then a patient may succumb to its cardiac complications, and of these, the post-mortem examinations have hitherto been limited to the immediate seat of the diseased structures, and notes made of their pathological condition, without even a thought of carrying the investigations beyond that point. And those who have become permanently deformed from the effects of the disease, usually live till the pathological condition of the cord gives place to the physiological; but from any changed condition of diseased structure, there is seldom or never a recovery. Pathologists have hitherto only directed their investigations to the local changes that have taken place in the diseased structure; hence we have ascertained, that, though the rheumatic inflammation has its primary seat in the fibrous tissue, and is not disposed to terminate in the usual manner of ordinary inflammations, yet when by contiguous sympathy it seizes upon the serous, muscular or other tissues, the programme of its termination is changed to those of other inflammations, and that, too, without in the least affecting the primary inflammation, or interfering with the original cause.

But there is a termination of pure rheumatic inflammation—if we may be permitted to call muscular atrophy a termination—in the condition known as atrophy, where all apparent signs of inflammation in the interstitial and muscular texture have subsided—provided such inflammation ever had an existence in fact, a matter, which to my mind is very questionable; because when a muscle, as is supposed, becomes implicated in rheumatic inflammation, the probabilities are, that the cellular tissue surrounding the muscle and every fibre and intricate cell-structure of the muscle, that this tissue is really, and in fact, the seat of the disease; the pain being caused by the movement or contractility of the muscular fibre, thus interfering with the quiet or passive condition of this tissue, the muscular fibre probably entirely exempt from the primary inflammation, but may, like other structures, become involved on account of the intricate connection of the structures. It may be mentioned, in this connection that atrophy is a condition not unfrequently resulting from rheumatic inflammation, and the cause is usually attributed entirely to the want of proper exercise; the pain produced by movement creating or causing the inability to use the muscles; hence from inactivity alone the absorbents are enabled to maintain a degree of activity sufficient to carry off the worn-out or broken down tissue faster than the recuperative powers of nature can build it up. But this reason is not sufficient of itself, to my mind, to account satisfactorily for the cause of atrophy, and while I am at once ready to admit, that inactivity of a particular muscle may be, under particular circumstances a sufficient cause of atrophy, yet when there is no "power behind the throne," as it were, to account for the inflammation that in rheumatism precedes the atrophy, there is nothing more certain in nature than that it would yield to appropriate local, general or specific treatment, and the atrophy be checked by exercise, friction, the electrical current, or by a spontaneous effort of nature to readjust the nutrition of the parts by vitalizing the cellulo-molecular formation to that extent that it predominates over the disintegration and absorption of the muscular tissue.

But is this the case where atrophy is the result of rheumatic inflammation? Does any local treatment avail for either the atrophy or inflammation to put a stop to their certain and alarming disintegration? The answer is, most emphatically, No. Then where are we to look for the primary cause unless it be in the functional derangement of the spinal cord—that great vitalizer of the whole animal organism? And where are we to seek a remedy, excepting among those agents, the action of which is directed to the spinal cord, by either direct general or specific action?

The same rule holds good where there is atrophy from any other cause. We have, for instance, a case of atrophy or paralysis, perhaps both, from compression of the cord, by the dorsal spine, from injury or other causes; these are incidents that necessarily arise from compression; or, there may be paralysis from inanition, as is the case in *infantile* paralysis, atrophy ensues, and, perhaps, in connection with this, there may be fatty degeneration of the tissues, which is not at all uncommon even after rheumatic inflammation of the cellulo-muscular tissues.

In this case—that of injury—do physicians institute any other treatment than to keep the extremities warm? Would not any professional man be considered wanting in acumen who would undertake to drug such a patient, with the expectation of benefiting him any further than by general or local antiphlogistic appliances he might prevent or lessen the local inflammation that would be likely to ensue? Would not the chief attention be paid to the local treatment? And if, by any surgical manipulation, the compression could be removed, would not that be resorted to as the primary means for the restoration of the spinal functions? And, when that was accomplished, nothing further would be required, except the ever-present *vis atergo* to restore to the limbs and muscles their proper functions.

And if this surgical interference should fail, and the powers of nature—the greatest of all panaceas for human ills—should prove abortive, or insufficient to restore the integrity of the functions of the spinal cord, we then have presented the same phenomena—atrophy with its concomitants—as in, so-called, muscular rheumatism, but without the pain of inflammation of the articular and cellular tissue. But herein consists the primary cause of atrophy in either case; it being produced from a want of equilibrium between the vital forces connected with the breaking down and building up, or the nutritive functions of the animal organism, which condition is brought about and sustained by a disturbance from one cause or another of the great center of organic life—the spinal cord.

Thus, we perceive the universality of the law—that like causes, from whatever source they may originate, produce the same effect, and in neither case would paralysis, proper, on the one hand, or paralysis from inertia on the other, be necessarily followed by atrophy, were it not, as formerly suggested, for the disturbance of the vital forces originating in spinal derangement, or absolute disease of this function.

Taking it for granted, therefore, that this hypothesis is correct, the action of the whole catalogue of anti-rheumatic remedies that experience has taught us is most reliable in the treatment of this disease, may be easily explained by the conceded theory of their action on the animal economy. Opium, for instance, manifests its curative agency in allaying pain, and producing rest, which is of the greatest therapeutic benefit in equalizing the circulation and rendering accumulations less likely to occur, and even removing such accumulations from the central organs, and lessening the pressure upon the capillaries of the medulla spinalis. Venesection manifests its agency by the immediate sedation produced upon the heart and arteries, the relaxation of the capillaries, exhalents and emunctories of the general system. The agency of the saline cathartics is manifested by lessening the amount of the circulating fluids by means of copious alvine evacuations, thereby directing, or rather changing the current of the circulation and lessening its accumulation in the nerve centres.

And now, since the pathology of this disease is somewhat better understood, and the theory of the acidity of the secretions maintained, including a superabundance of fibrin in the blood as ascertained by our most accurate observers, it is probable that the alkalies manifest their remedial agency through the neutralization of the acids, as well as their tendency to lessen the production of fibrin in the blood, hence lessening the liability to the formation of false membranes upon the free surfaces attacked by rheumatic inflammation.

Colchicum, probably, manifests its curative agency in more points than any other single remedy in this harassing disease, and each particular point would be found to possess more or less of a direct relation to either lessening or relieving local congestions, such, for instance, as its known direct action upon the kidneys and the cutaneous exhalents, producing copious diuresis on the one hand or profuse perspiration on the other; besides, after it has begun to bring the emunctories of the system under its influence, among its manifestations are recognized those of sedation, affecting the impulse of the heart and arteries, and at the same time producing copious discharges from the bowels. These are only a portion of its recognized effects, and in this place we will not speculate upon its probable direct or specific influence over rheumatic inflammation, which is of no small consideration judging from its long popularity with the profession, as a remedy in rheumatic inflammation.

We might mention other remedies used under the varied condition of circumstances presented by this disease, among which are guaiac. and gelseminum, and assign to each its place, and point out the particular and distinct position that they each maintain in the treatment of this disease, but would probably fail to throw any material light upon the subject; therefore, from what has been said, and the conclusions we have drawn, there can be nothing more simple than what the plain indications of treatment are, and what should be apparent to every one.

It is simply cupping, or blistering the spine as circumstances may require, in conjunction with the use of local and general remedies, such as the judgment of the physician in each particular case may dictate. We began this treatment as early as 1856, immediately after hearing Prof. Mitchell deliver his course on rheumatism, and in no instance, in a very extensive country practice, has this method disappointed our most sanguine expectations; neither has it made any difference in the application of cups to the spine, whether the disease was in the acute, sub-acute or chronic form, for this, in our opinion, is one of the first, last and main indications in its successful treatment, and therefore no time is to be lost in dallying with other means, but immediately resort to dry cupping with as large glasses as can be borne by the patient, and should circumstances be such that in our judgment a resort to vesication, or if only irritants or rubefacients, would be preferable, it is attended to at once, and in either case with the greatest relief to the suffering patient. There has been no instance of this disease under our care that has not speedily yielded to this treatment, along with appropriate internal and local remedies, and, indeed, much more readily than formerly when no attention was paid to the spine.

Among the constitutional remedies are those previously mentioned, but above all others stand colchicum and bi-carb. potassa. Locally, cotton batting, friction with rubefacients as the necessities of the case may require, may be applied to the affected parts. The average time required, in our practice, in a given number of cases, to place our patients on the convalescent list under this treatment, is from ten to twelve days; whereas under the ordinary practice, where no attention is paid to the spine, the average duration of the disease, in a like number of cases, is from sixteen to twenty days. Another thing that is worthy of note, in this connection, and which is of no small interest, both to the professional man and the patient, is, that since we have adopted this practice we have never seen a single instance of either endocarditis or pericarditis occur among our rheumatic patients, and besides the difference in the time of convalescence gained by our present practice, our patients have all had a more speedy and perfect recovery; and in no instance has the disease manifested so migratory a tendency as is usual, leaving the heart entirely exempt from its unfriendly visitations; and should further experience go to establish the fact, that cupping or blistering the spine, renders rheumatic inflammation less migratory, it will at least strip the disease of one of its greatest sources of danger, and give us more confidence in giving a prognosis in a given case.

During the two years that we were in the army as surgeon of the 56th Regiment Illinois Vol. Infantry, from 1861 to 1863 we had no disease that gave us so much trouble and annoyance as rheumatism in all its forms, excepting perhaps diarrhœa and dysentery. Its most prevalent forms were lumbago and intercostal, from their mildest forms to their most distressing character. Our government supplies at best were meager enough, and we would often run out of a leading article for particular diseases long before we would have an opportunity of making a new requisition. At our morning sick-call, on almost every day, many of this character of cases would present themselves for treatment; as a matter of course, our acet. ext. of colchicum could not go very far, as we were only permitted to draw a certain amount for a given time, and hence many of our army surgeons who cared well for the sick under their charge, were more than half the time out of this important drug, and were, therefore, borrowers from those who cared less for those under their charge, or were ignorant of what was so often absolutely necessary for the welfare of their men. But by paying attention to cupping the spine, in the secret of which we had our assistant surgeon, and hospital corps thoroughly informed, we managed to get along admirably with our meager governmental supply of colchicum, made into pills with ipecac. Of these we ordered three each day, with from three to four twenty-grain doses of bi-carb. soda. By these means we were enabled to keep our men on duty during the whole time of treatment; whereas, under ordinary circumstances they would have remained on the sick list and finally become afflicted, as thousands did, with nostalgia, and either died, been discharged, or remained an incubus upon the government while they continued in the service.

Many of our regiment would come to me at the hour of sick-call, and say, "I do not want to be put on the sick-list, but if you will order me cupped, as I was yesterday, I shall be able for duty, and get along admirably, as the cupping yesterday cured me of my aches and pains."

This was our experience in the army, and it has been the same in private practice, in regard to this treatment, for sixteen years; and, therefore, for the benefit of suffering humanity I would most earnestly recommend it for adoption to my professional brethren. And if, in the progress of their professional inquiries, they should ascertain as a fact, that there is a "balm in Gilead," I trust that they will not hesitate, either from prejudice or conceit, to use it to allay the pangs of suffering humanity; because, it is no small matter to our patients to be relieved of from two to six weeks of the most agonizing suffering, to say nothing of the advantages to be derived from a more perfect restoration to health than is usually obtained under the ordinary means of cure.

